# A Combination of Chemotherapy and Oncolytic Virotherapy Sensitizes Colorectal Adenocarcinoma to Immune Checkpoint Inhibitors in a cDC1-Dependent Manner

**DOI:** 10.3390/ijms23031754

**Published:** 2022-02-03

**Authors:** Nader El-Sayes, Alyssa Vito, Omar Salem, Samuel Tekeste Workenhe, Yonghong Wan, Karen Mossman

**Affiliations:** 1 McMaster Immunology Research Centre, Department of Medicine, McMaster University, Hamilton, ON L8S 4K1, Canada; elsayesn@mcmaster.ca (N.E.-S.); salemo@mcmaster.ca (O.S.); wanyong@mcmaster.ca (Y.W.); 2 Faculty of Health Sciences, McMaster University, Hamilton, ON L8S 4K1, Canada; 3 Department of Clinical Translation, Ontario Institute for Cancer Research, Toronto, ON M5G 0A3, Canada; alyssavito87@gmail.com; 4 Department of Pathobiology, Ontario Veterinary College, University of Guelph, Guelph, ON N1G 2W1, Canada; sworkenh@uoguelph.ca

**Keywords:** colorectal cancer, immunotherapy, immune checkpoint inhibitors, oncolytic virus, chemotherapy

## Abstract

Immune checkpoint therapy has shown great promise in the treatment of cancers with a high mutational burden, such as mismatch repair-deficient colorectal carcinoma (dMMR CRC). However, many patients fail to respond to immune checkpoint therapy. Using a mouse model of dMMR CRC, we demonstrated that tumors can be further sensitized to immune checkpoint therapy by using a combination of low-dose chemotherapy and oncolytic HSV-1. This combination induced the infiltration of CD8^+^ and CD4^+^ T cells into the tumor and the upregulation of gene signatures associated with the chemoattraction of myeloid cell subsets. When combined with immune checkpoint therapy, the combination promoted the infiltration of activated type 1 conventional dendritic cells (cDC1s) into the tumor. Furthermore, we found this combination strategy to be dependent on cDC1s, and its therapeutic efficacy to be abrogated in cDC1-deficient Batf3^−/−^ mice. Thus, we demonstrated that the adjuvanticity of dMMR CRCs can be improved by combining low-dose chemotherapy and oncolytic HSV-1 in a cDC1-dependent manner.

## 1. Introduction

Colorectal cancer (CRC) accounts for roughly 10% of cancer-related deaths worldwide [1]. Even with early detection, 25–50% of patients with early-stage CRC develop metastatic disease [2]. Immune checkpoint inhibitors (ICIs) have shown tremendous promise in the treatment of solid tumors with high mutational burdens, such as melanoma and lung cancer. To this end, mutational burden has been utilized as a biomarker for ICI therapy for multiple types of cancer [3,4]. CRC can be classified into two groups based on deficiencies in mismatch repair and microsatellite instability. Mismatch repair-deficient (dMMR) tumors have higher mutational burden, which makes them good candidates for ICI therapy. Indeed, ICI therapy has shown great promise in dMMR CRC, with close to 40% of patients demonstrating an overall response to pembrolizumab [5]. While this level of response is promising, many patients with dMMR CRC do not respond to ICI therapy. Therefore, additional studies are required to identify factors in the tumor microenvironment (TME) that can enable a response to ICI therapy in dMMR CRCs. 

While mutational burden and antigenicity are potential biomarkers for ICI therapy, several other factors can influence the response to therapy. Namely, adjuvanticity is required to promote infiltration and activation of immune cells at the tumor site. Immunogenic cell death (ICD) can improve adjuvanticity through the release of DAMPs and other danger signals [6]. To increase adjuvanticity and sensitize tumors to ICI therapy, several combination strategies have been developed to induce ICD [7]. These include the combination of ICI therapy with clinically relevant chemotherapies and emerging therapies such as oncolytic virus (OV) therapy [8,9]. While chemotherapy can be immunosuppressive with dose-dense regimens, several chemotherapeutic agents have demonstrated immunogenic properties when used at lower doses [10]. Our group has previously utilized combinations of oncolytic HSV-1 (oHSV) and low-dose chemotherapies to sensitize breast adenocarcinomas to ICI therapy [11,12]. We further demonstrated that low-dose mitomycin C (mito) combined with oHSV improves the susceptibility of tumors to ICI therapy through induction of necroptosis [12]. While these combinations are effective in sensitizing tumors to ICI therapy, there remains a fundamental lack of understanding of the changes in the TME that improve adjuvanticity and drive therapeutic outcomes. 

In this study, we investigated the combination of mito + oHSV in MC38 tumors, a murine model of dMMR CRC with high microsatellite instability [13]. We found that while mice harboring MC38 tumors moderately responded to ICI therapy, they did not maintain durable responses. Addition of mito + oHSV was successful in further sensitizing tumors to ICI therapy, resulting in durable responses in 55% of mice. The combination of mito + oHSV + ICI induced inflammation id the TME and promotes the recruitment of myeloid cell subsets. In particular, type 1 conventional DCs (cDC1s) showed high levels of tumor infiltration after treatment. Finally, we demonstrated that the therapeutic efficacy driven by mito + oHSV + ICI was dependent on the presence of cDC1s. Altogether, these data show that therapeutic outcomes to ICI therapy can be further improved in patients with dMMR CRC with the addition of combinations that improve tumor adjuvanticity. Furthermore, we demonstrated that enabling a response to ICI therapy in this model was dependent on the cDC1 subset. This is in line with other reports showing a crucial role of cDC1s in enabling a therapeutic response to ICI therapy [14,15].

## 2. Results

### 2.1. A Combination of Low-Dose Mitomycin C and Oncolytic HSV-1 Sensitizes Colon Adenocarcinoma Tumors to ICI Therapy

**Figure 1:**
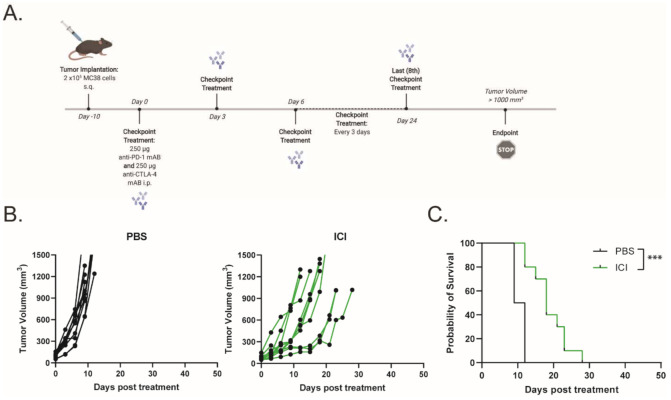
Immune checkpoint inhibitor therapy exhibits moderate response against MC38 tumors. (**A**) Schematic representation of the ICI treatment regimen. (**B**) Tumor growth kinetics and (**C**) Kaplan–Meier survival curves for MC38 tumor-bearing mice treated with saline or ICI. Event occurrence in Kaplan–Meier survival curves indicates endpoints based on tumor volumes. *** = *p* < 0.001.

**Figure 2:**
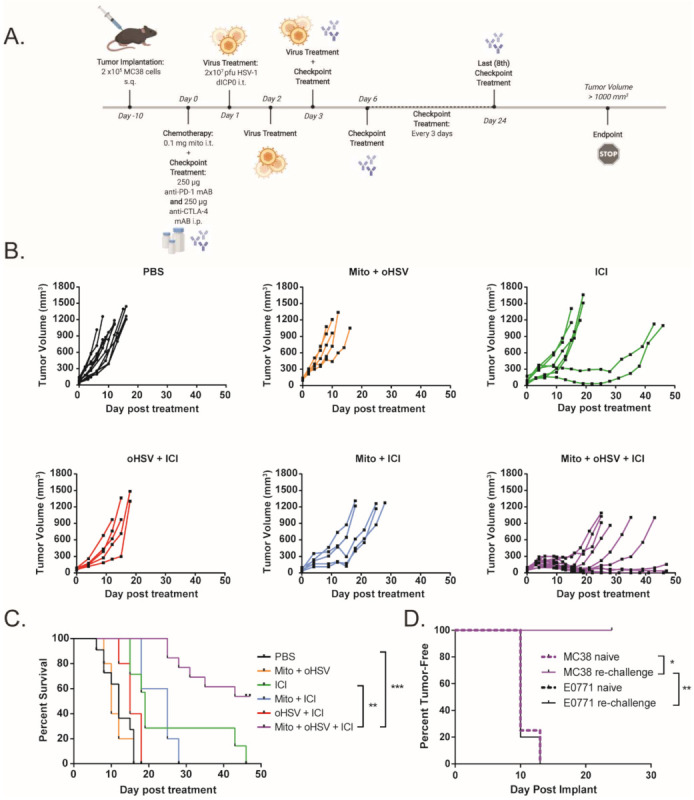
A combination of mitomycin and oHSV sensitizes MC38 tumors to ICI therapy. (**A**) Schematic representation of the combination treatment regimen. (**B**) Tumor growth kinetics and (**C**) Kaplan–Meier survival curves for MC38 tumor-bearing mice treated with different combinations of mito, oHSV, and/or ICI. (**D**) Mice that had a complete response to mito + oHSV + ICI treatment were re-challenged with either MC38 or E0771 tumors, and the percent of tumor-free mice was graphed as a Kaplan–Meier curve. Age-matched naïve mice were used as controls for tumor challenge. Event occurrence in Kaplan–Meier survival curves indicates endpoints based on tumor volumes. * = *p* < 0.05, ** = *p* < 0.01, *** = *p* < 0.001.

To assess the efficacy of ICIs (dual anti-PD-1 and anti-CTLA-4 monoclonal antibodies) in a murine model of dMMR CRC, C57BL/6 mice bearing MC38 tumors were treated with 250 μg of anti-PD-1 and anti-CTLA-4 monoclonal antibodies every 3 days for a total of eight doses, while monitoring tumor growth (Figure 1A). Tumor-bearing mice demonstrated a partial response to ICI therapy, with delayed tumor progression and prolonged overall survival (Figure 1B,C). However, none of the mice demonstrated a durable response to ICI therapy. This finding is consistent with other reports that showed a moderate response to ICI therapy in MC38 tumors [16,17,18]. To increase the immunogenicity of the MC38 tumors and sensitize the further tumors to ICI therapy, we treated the mice with a combination of low-dose mito and oHSV. MC38 tumor-bearing mice were treated with a therapeutic regimen consisting of mito, oHSV, and/or ICI (Figure 2A). The combination of mito + oHSV showed no delay in tumor progression or survival benefit in the MC38 tumor model and Figure 2B). Similarly, the combinations of mito + ICI and oHSV + ICI demonstrated no improvement over ICI therapy; however, the full combination of mito + oHSV + ICI resulted in initial tumor regression in 100% of mice and a durable response in 54% of mice (Figure 2B,C). 

To assess the generation of persistent memory against MC38 tumors, mice that achieved a complete response to therapy were rechallenged with MC38 CRC or E0771 breast carcinoma cells. While all mice challenged with E0771 cells developed palpable tumors within 15 days, all mice challenged with MC38 cells inhibited tumor growth (Figure 2D). These data suggest that mito + oHSV therapy is not sufficient for therapeutic efficacy in MC38 tumors but can enable a durable tumor-specific response to ICI therapy.

### 2.2. Mito + oHSV + ICI Induces Tumor Infiltration of T Cells and Is Dependent on T Cells for Tumor Control

**Figure 3:**
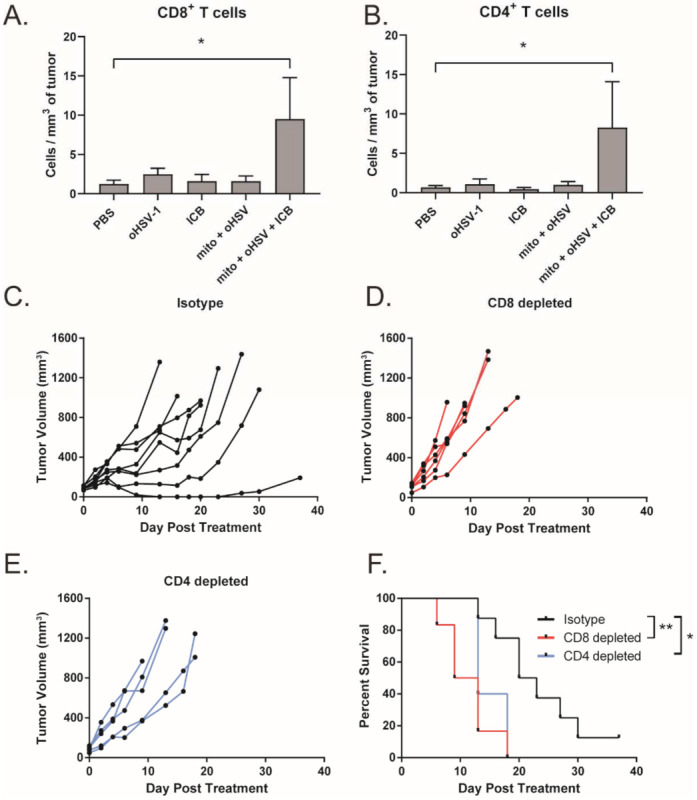
Efficacy of the mito + oHSV + ICI combination is dependent on CD8^+^ and CD4^+^ T cells. (**A**,**B**) Mice bearing MC38 tumors were treated with mito, oHSV, and/or ICI. Tumors were harvested 7 days after the final day of treatment, and the infiltration of CD8 and CD T cells was assessed by flow cytometry. (**C–E**) Tumor growth kinetics from tumor-bearing mice that were treated with mito + oHSV + ICI before the administration of CD8 and CD4 depletion antibodies. (**F**) Kaplan–Meier survival curves for anti-CD8, anti-CD4, or isotype antibody treated mice. Event occurrence in Kaplan–Meier survival curves indicates endpoints based on tumor volumes. * = *p* < 0.05, ** = *p* < 0.01.

We have previously shown that the combination of mito + oHSV can induce CD8^+^ T cell tumor infiltration in breast adenocarcinoma [12]. Given that mito + oHSV fails to control the growth of MC38 tumors in the absence of ICIs, we investigated the level of T cell infiltration across treatment groups. Interestingly, we found that mito + oHSV did not improve the infiltration of T cells into the tumors. However, the addition of ICIs significantly improved the infiltration of CD8^+^ and CD4^+^ T cells, despite the inability of ICI alone to induce T cell infiltration (Figure 3A,B). 

To further characterize the importance of T cells in mediating a therapeutic response to mito + oHSV + ICI, T cells were depleted using anti-CD8 and anti-CD4 monoclonal antibodies. Depletion of CD8^+^ and CD4^+^ T cells was confirmed by flow cytometry (Figure S2). We found that depletion of either CD8^+^ or CD4^+^ T cells abrogated tumor control and survival benefits mediated by mito + oHSV + ICI therapy (Figure 3C–F). This outcome is consistent with our previous findings in breast adenocarcinoma models [12]. 

### 2.3. The Mito + oHSV Combination Induces a Transcriptome Signature Associated with Myeloid Cell Recruitment and Activation

**Figure 4:**
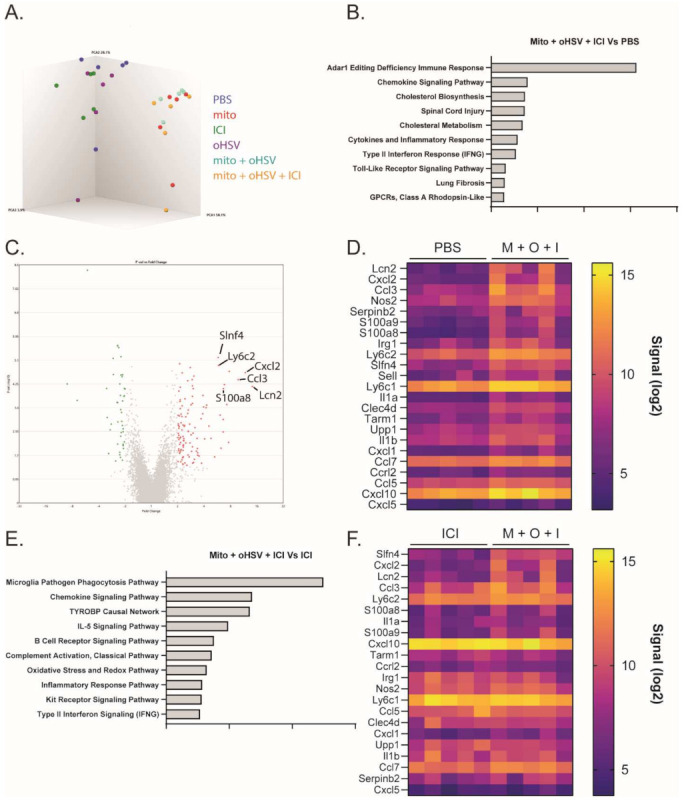
The combination mito + oHSV + ICI induces RNA transcriptomes associated with the recruitment and activation of myeloid subsets. Mice harboring MC38 tumors were treated with different combinations of mito, oHSV, and/or ICI. RNA was harvested from the tumors one day after the final treatment and sent for analysis by Clariom S assay. (**A**) 3-D cluster plot showing the RNA expression correlations between the different groups. (**B**) Pathway enrichment analysis showing the top 10 signaling pathways differentially expressed by the mito + oHSV + ICI group compared to the PBS controls. (**C**) Volcano plot and (**D**) heat map showing genes differentially expressed in mito (M) + oHSV (O) + ICI (I) groups compared to the PBS control. (**E**) Pathway enrichment analysis showing the top 10 signaling pathways differentially expressed in the mito + oHSV + ICI group compared to the ICI group. (**F**) Heat map showing genes differentially expressed in the mito + oHSV + ICI group compared to the ICI group.

The combination of mito + oHSV could enable a durable response to ICI therapy in MC38 tumors; however, the combination was insufficient to generate a response in the absence of ICIs. To better characterize relevant changes in the TME that can enable a durable response to ICI therapy, we compared changes in the transcriptomes of the treated mice. To this end, RNA was harvested from the tumors one day after the final treatment with mito, oHSV, and/or ICI for analysis using a Clariom S assay. Principal component analysis showed that all groups involving treatment with mito clustered together, despite a lack of therapeutic efficacy with mito monotherapy or mito + ICI combination therapy (Figure 4A). Pathway enrichment analysis identified several pathways that were upregulated in the mito + oHSV + ICI groups compared to PBS controls (Figure 4B). Of particular interest were pathways associated with chemokine signaling, inflammatory response, type II interferon signaling, and toll-like receptor signaling. Further in-depth analysis revealed upregulation of genes associated with the recruitment, maturation, and activation of myeloid subsets (Table 1 and Table S1, Figure 4C,D). The same gene signature was upregulated in mito + oHSV groups relative to PBS controls (Table 1 and Table S1, Figure S3). Furthermore, several of these signaling pathways and genes were upregulated in mito + oHSV + ICI groups compared to ICI alone groups (Table 1, Figure 4E,F). In particular, mito + oHSV + ICI induced the upregulation of genes involved in DC recruitment, activation, and antigen presentation compared to ICI alone. These data suggest that the combination of mito + oHSV induces tumor infiltration and activation of DCs and other myeloid cell subsets, thereby sensitizing MC38 tumors to ICI therapy. 

### 2.4. Mito + oHSV Induces Tumor Infiltration of cDC1 Subsets and Is Dependent on Batf3

**Figure 5:**
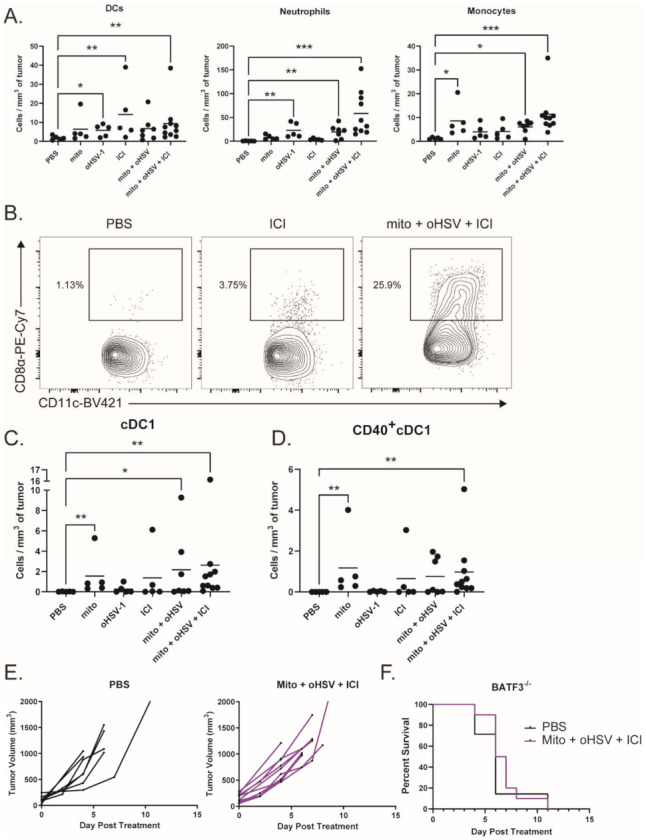
The combination mito + oHSV + ICI promotes tumor infiltration of cDC1s. (**A**) Mice bearing MC38 tumors were treated with different combinations of mito, oHSV, and/or ICI. Tumors were harvested 4 days after the start of treatment, and the frequency of infiltrating immune cells was analyzed by flow cytometry. DCs (CD11c^+^ MHCII^+^), neutrophils (CD11b^+^ Ly6C^mid^ Ly6G^+^), and monocytes (CD11b^+^ Ly6C^hi^ Ly6G^-^) were graphed. (**B**) Representative flow plots of CD8^+^ DCs (cDC1s). (**C**) Graphs of tumor-infiltrating cDC1s and (**D**) CD40^+^cDC1s, (**E**) BATF3^−/−^ mice harboring MC38 tumors were treated with PBS or mito + oHSV + ICI. Tumor volumes and (**F**) Kaplan–Meier survival curves were graphed. Event occurrence in Kaplan–Meier survival curves indicates endpoints based on tumor volumes. * = *p* < 0.05, ** = *p* < 0.01, *** = *p* < 0.001.

To characterize myeloid subsets in the tumor, we treated tumor-bearing mice with combinations of mito, oHSV, and/or ICI before harvesting the tumors on day 4 of treatment. Tumor infiltrates were then characterized by multicolor flow cytometry. We found that several subsets of myeloid cells infiltrated the tumor after treatment with mito + oHSV (Figure 5A). Interestingly, the treatment with mito induced the infiltration of monocytes (CD11b^+^ Ly6C^hi^ Ly6G^-^), while the treatment with oHSV induced the infiltration of neutrophils (CD11b^+^ Ly6C^hi^ Ly6G^-^) and DCs (CD11c^+^ MHCII^+^). In all three cases, however, the full combination of mito + oHSV + ICI induced the largest number of tumor infiltrates. 

Given the role of DCs in antigen presentation, we further characterized the DC subsets infiltrating the tumor. The priming of antitumor T cells is dependent on the cross presentation of tumor antigens by cDC1s [15,43,44]. Interestingly, the treatment with mito induced the infiltration of cDC1s (CD8α^+^ DCs) into the tumor, which was further increased by the addition of oHSV (Figure 5B,C). However, the treatment with mito + oHSV + ICI induced the greatest level of cDC1 infiltration into the tumor. Additionally, the treatment with mito + oHSV + ICI induced the highest level of cDC1 activation, characterized by their expression of CD40 (Figure 5D). In contrast, type 2 conventional DC (cDC2) infiltration was improved by ICI therapy, but not by the full combination of mito + oHSV + ICI (Figure S4). The infiltration of monocyte-derived DCs (moDCs) was also improved in all groups treated with mito or ICI (Figure S4); however, the role of moDCs in cancer immunotherapy is still under debate [45,46]. 

To establish the relevance of cDC1 tumor infiltration in enabling a therapeutic response to ICI therapy, we used Batf3^−/−^ mice which are deficient for cDC1s [47]. We found that treatment of tumor-bearing Batf3^−/−^ mice with mito + oHSV + ICI was ineffective in controlling tumor growth or prolonging survival (Figure 5E,F). Altogether, these data suggest that the recruitment and activation of cDC1s are required for mito + oHSV + ICI-mediated tumor control. 

## 3. Discussion

In this study, we demonstrated that the response to ICI therapy can be improved by utilizing a combination of mito + oHSV in a murine model of dMMR CRC. In the absence of ICIs, mito + oHSV fails to control tumor growth or prologue the survival of tumor-bearing mice. Although we previously demonstrated improved infiltration of T cells in murine models of breast adenocarcinoma after treatment with mito + oHSV [12], this combination was ineffective in improving T cell infiltration in CRC tumors in the absence of ICI therapy. This outcome is likely a result of the immunosuppressive nature of MC38 tumors, which have been reported to maintain high levels of PD-L1 expression that contribute to immune evasion [48]. However, the addition of ICIs resulted in a significant increase in T cell infiltrates. These data suggest that the combination of mito + oHSV can further improve the response to ICI therapy. Indeed, several clinical trials are underway in dMMR CRCs combining chemotherapy with ICIs [2]. 

Despite having no therapeutic efficacy in MC38 tumors, mito + oHSV induced the upregulation of genes associated with the recruitment of myeloid subsets. Furthermore, the combination induced the infiltration of DCs, neutrophils, and monocytes into the tumor. This observation is in line with our previous work in which we found chemokine signatures associated with myeloid cells in breast adenocarcinoma after treatment with mito + oHSV [12]. Of particular interest was the improved infiltration of cDC1s into MC38 tumors, which are required for priming endogenous tumor-specific T cells and enabling the response to ICI therapy [47]. We found that the response to mito + oHSV + ICI was abrogated in Batf3^−/−^ deficient for cDC1s. Indeed, other reports have shown that strategies to promote the infiltration and activation of cDC1s can sensitize tumors to immunotherapy [14,15]. For example, one study led by Salmon et. al. demonstrated that the combination of FLT3L and poly I:C can improve the expansion and activation of cDC1s, leading to improved priming of antitumor T cells and a better response to ICI therapy [14]. Similarly, FLT3L and poly-ICLC enhanced cDC1-mediated cross priming and synergized with anti-CD137 and anti-PD-1 therapy in MC38 tumors [15]. It should be noted, however, that in Batf3 knockout mice there may be unspecific effects on other immune cells. While mito + oHSV induced the infiltration of cDC1s in the absence of ICI therapy, the addition of ICIs resulted in a consistently elevated infiltration of activated cDC1s. Interestingly, one report has demonstrated that CD40 expression in DCs is inhibited in MC38 tumors [49]. Furthermore, PD-1 expression in DCs was shown to dampen their activation [50]. These findings suggest that the addition of ICIs can improve the activation of cDC1s, while mito + oHSV improves cDC1 infiltration into the tumor. Indeed, the full combination of mito + oHSV + ICI is the only treatment that significantly improved the infiltration of CD8^+^ and CD4^+^ T cells. While CD4^+^ T cells are MHC-II restricted, cDC1-mediated priming of CD4^+^ T cells is required for optimal antitumor activity [43]. These reports are in line with our results showing that the depletion of CD4^+^ T cells abrogates tumor control by mito + oHSV + ICI. Future studies should assess the ability of mito + oHSV to promote cDC1 maturation in the tumor and improve MHC-I- and MHC-II-mediated antigen presentation by cDC1s.

While this study focused on the infiltration of cDC1s, mito + oHSV induced the infiltration of several other myeloid subsets, including neutrophils. The role of tumor-associated neutrophils (TANs) in immunotherapy has been highly controversial. N1 TANs can exert antitumor activity through direct and indirect cytotoxicity, while N2 TANs are widely associated with immunosuppression and metastasis. It is currently unclear whether the induction of TAN infiltration by mito + oHSV + ICI is beneficial, detrimental, or irrelevant for therapeutic efficacy. We also found that ICI therapy could induce the infiltration of cDC2s into the tumor, which was decreased by the addition of mito + oHSV. Future work should assess the potential of mito + oHSV to promote the differentiation of pre-DCs into cDC1s rather than cDC2s. 

ICI therapy has demonstrated most success in solid tumors with high mutational burden, such as melanoma, lung cancer, and dMMR CRC [3,4,5]. However, the majority of patients still fail to respond to ICI therapy, which highlights the need for improvement. Recent preclinical reports suggest that increasing tumor adjuvanticity through ICD-inducing therapies can enable better responses to ICI therapy [6,7]. We showed that the combination of low-dose mitomycin C and oncolytic HSV-1 can enable the response to ICI therapy in a cDC1-dependent manner in dMMR CRC. We believe that combination therapies that can induce ICD have the potential to improve tumor adjuvanticity, which, in turn, can improve therapeutic outcomes in cancers with sufficient antigenicity. 

## 4. Material and Methods

### 4.1. Cell Lines

MC38 cells (ATCC) were maintained in Dulbecco’s modified Eagle’s medium (DMEM) supplemented with 5% fetal bovine serum (FBS, ATCC 30-2020), 2 mmol/l L-glutamine, 100 U/mL penicillin, 100 µg/mL streptomycin (Gibco). 

### 4.2. Virus Propagation

HSV-1Δ810 (oHSV) is an oncolytic attenuated variant of HSV-1 with a deletion in the ICP0 region. The virus was propagated, purified, and quantified in U2OS cells as described previously [51]. 

### 4.3. In Vivo Experiments

Mice were maintained at the McMaster University Central Animal Facility, and all the procedures were performed in full compliance with the Canadian Council on Animal Care and approved by the Animal Research Ethics Board of McMaster University. MC38 tumors: 2 × 10^5^ cells were implanted subcutaneously into the left flank of 6–8-week-old female C57/Bl6 mice (Charles River Laboratories, Wilmington, MA, USA). On the first day of treatment, 0.1 mg of Mitomycin C (Sigma-Aldrich, Saint Louis, MO, USA), 250 µg of αPD-1 and αCTLA-4 (InVivoMab, Lebanon, NH, USA) antibodies were administered by i.t. and i.p. injections, respectively. For the following 3 days, 2 × 10^7^ pfu of oHSV was administered by i.t. injection (total of 3 doses). Experimental groups receiving αPD-1/CTLA-4 followed a dosing schedule of 250 µg treatments every 3 days for a total of 8 doses. For T cell depletions, 250 µg of αCD8 or αCD4 antibodies were administered by i.p. injection once per week, starting the day before treatment and continuing until the endpoint. Tumor volumes were monitored and measured every 2–3 days until they reached their endpoint volume (1000 mm^3^). 

### 4.4. Immune Analysis and Flow Cytometry

Tumors were harvested on days 4 and 7 of treatment before being processed. The tumors were diced into fine pieces, then subject to digestion using Liberase (Sigma-Aldrich), as described in the manufacturer’s protocol. The digested tumors were then passed through a 100 µm cell strainer. Red blood cells were lysed with ACK buffer, and the remaining cells were transferred to a round-bottom 96-well plate. The cell suspensions were stained with fixable viability stain 510 (BD Biosciences, Mississauga, ON, Canada) for 30 min at room temperature, then treated with anti-CD16/CD32 antibodies (Fc block; BD Biosciences) for 15 min at 4 °C. Cell surface staining was done for 30 min at 4 °C. Intracellular staining was performed using the cytofix/cytoperm Fixation/Permeabilization kit (BD Biosciences). Data acquisition was done on the LSRFortessa (BD), and data were analyzed using FlowJo. 

### 4.5. Clariom S Assay

Tumors were harvested one day after the final treatment and homogenized in Trizol. RNA was extracted using the RNeasy Plus Mini Kit (Qiagen, Hilden, Germany) according to the manufacturer’s protocol. RNA was reverse-transcribed, and cDNA was purified via magnetic beads and fragmented using UDG. Fragmented cDNA was then hybridized to the Affymetrix Clariom S mouse arrays (Thermo Fisher Scientific, Bedford, MA, USA), and the stained arrays were scanned to generate intensity data. Raw data were analyzed using the Thermo Fisher Transcriptome Analysis Console software. 

### 4.6. Statistical Analysis

Results are presented as means ± standard deviation. Log-rank (Mantel-Cox) tests were used to analyze the statistical significance of differences between treatment groups for Kaplan–Meier survival graphs. Ordinary one-way ANOVA was used to determine the statistical significance of differences between means of treated groups according to the normality of their distributions. In all cases, the null hypothesis was rejected when *p* values < 0.05. All statistical analyses were performed using GraphPad Prism 9.

## Figures and Tables

**Table 1:** Differentially expressed genes associated with myeloid subset recruitment and activation. M = mito, O = oHSV, I = ICI.

Gene Symbol	M + O + I vs PBS	M + O vs PBS	M + O + I vs I	Function
Lcn2	14.18	11.69	5.62	Expressed by DCs, contributes to antigen presentation and CD8 T cell priming [19,20].
Cxcl2	11.77	23.53	4.99	Expressed by activated DCs [21]. Involved in chemoattraction of neutrophils [22].
Ccl3	9.78	16.41	3.09	Enhances recruitment of cDC1s and T cells to the tumor. Enhances priming and proliferation of antitumor T cells [23].
Nos2	7.73	4.83	1.76	Expressed by activated DCs [24]. Expressed by M1 macrophages [25].
Serpinb2	7.55	3.04	3.22	Expressed by conventional DCs and macrophages [26].
S100a9	6.76	5.87	3.73	Expressed by DCs, neutrophils, and macrophages [27]. Promotes inflammation through TLR4 and RAGE signaling [28].
S100a8	6.62	7.54	2.81	Expressed by DCs, neutrophils, and macrophages. Promotes inflammation through TLR4 and RAGE signaling.
Irg1	6.6	3.57	−1.57	Marker of myeloid cells [29].
Ly6c2	5.81	5.08	1.45	Marker of myeloid cells [30].
Slfn4	5.8	7.46	4.18	Involved in differentiation of myeloid cells [31].
Sell	5.24	5.23	2.58	Regulator of leukocyte adhesion [32].
Ly6c1	4.26	2.66	1.31	Marker of myeloid cells [30].
Il1a	4.21	4.15	1.79	Involved in DC activation, facilitates T cell priming [33].
Clec4d	3.85	3.54	1.19	Expressed by neutrophils and monocytes [34].
Tarm1	3.69	1.91	−1.36	Expressed by DCs, neutrophils, and macrophages. Enhances secretion of proinflammatory cytokines [35].
Upp1	2.99	2.08	−1.32	Associated with antigen-presenting myeloid cells [36].
Il1b	2.96	2.24	1.69	Involved in DC activation, facilitates T cell priming [33].
Cxcl1	2.92	1.84	1.77	Involved in neutrophil chemoattraction [37].
Ccl7	2.74	1.88	2.32	Involved in chemoattraction of immune cells [38].
Ccrl2	2.63	2.53	−1.06	Expressed by neutrophils [39].
Ccl5	2.47	3.11	1.42	Involved in chemoattraction of DCs [40].
Cxcl10	2.41	2.18	−1.47	Expressed by cDC1s, induces recruitment of T cells [41].
Cxcl5	2.01	1.38	1.07	Involved in neutrophil chemoattraction [42].

## Data Availability

Clariom S assay data (Figure 4, Figure S3, Table 1 and Table S1) can be found in the GEO database (GSE195924).

## Supplementary Materials

The following are available online at https://www.mdpi.com/article/10.3390/ijms23031754/s1.
