# Supplementary material for: A Combination of Chemotherapy and Oncolytic Virotherapy Sensitizes Colorectal Adenocarcinoma to Immune Checkpoint Inhibitors in a cDC1-Dependent Manner
Source: Int J Mol Sci. 2022 Feb 3;23(3):1754. doi: 10.3390/ijms23031754 (PMC8915181; doi:10.3390/ijms23031754)
Supplement: Supplementary file 1 [file ijms-23-01754-s001.zip › ijms-1535608-SI.pdf]

International Journal of
Molecular Sciences

MDPI

Supplementary Figures

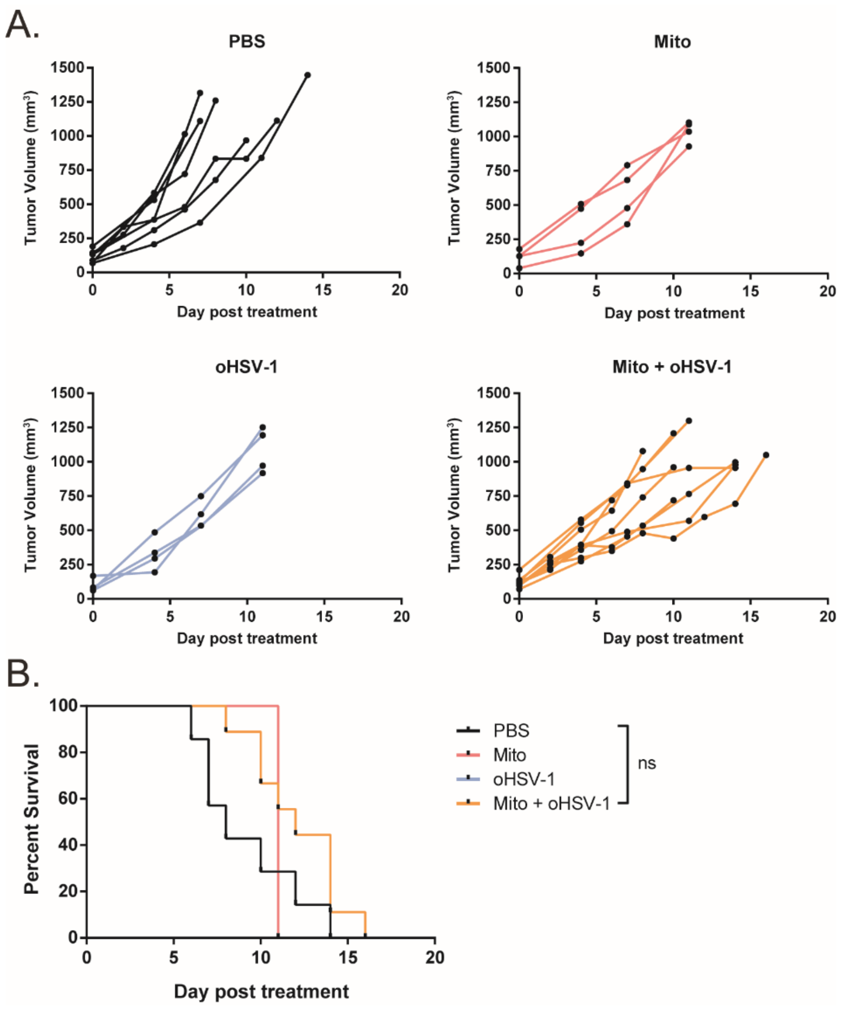

Figure S1. Combination of mito and oHSV fails to control MC38 tumors. (A) Tumor growth kinetics and **(B)** Kaplan-Meier survival curves from mice bearing MC38 tumors treated with different combinations of mito and oHSV monotherapy and combination therapy.

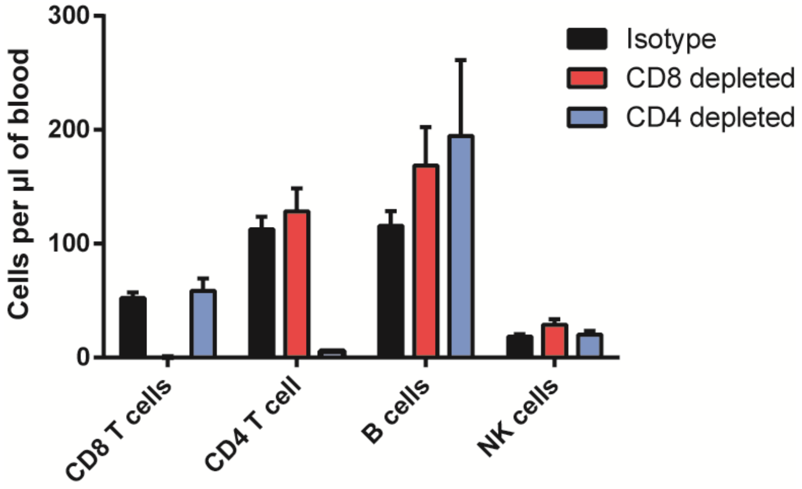

Figure S2. Depletion of T cells with monoclonal antibodies. Anti-CD8 or anti-CD4 monoclonal antibodies were administered to tumor-bearing mice by i.p. Number of CD8 T cells, CD4 T cells, B cells and NK cells in circulation were assessed by flow cytometry.

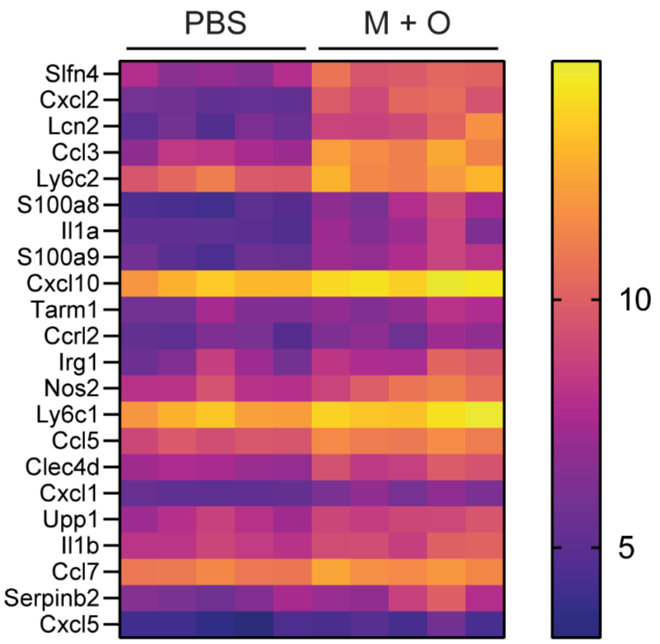

Figure S3. mito + oHSV induces RNA transcriptomes associated with recruitment and activation of myeloid subsets. Mice harboring MC38 tumors were treated with different combinations of mito, oHSV and/or ICI. RNA was harvested from the tumors one day after the final treatment and sent for analysis by Clariom S assay. The heat map shows genes differentially expressed between mito + oHSV compared to PBS control.

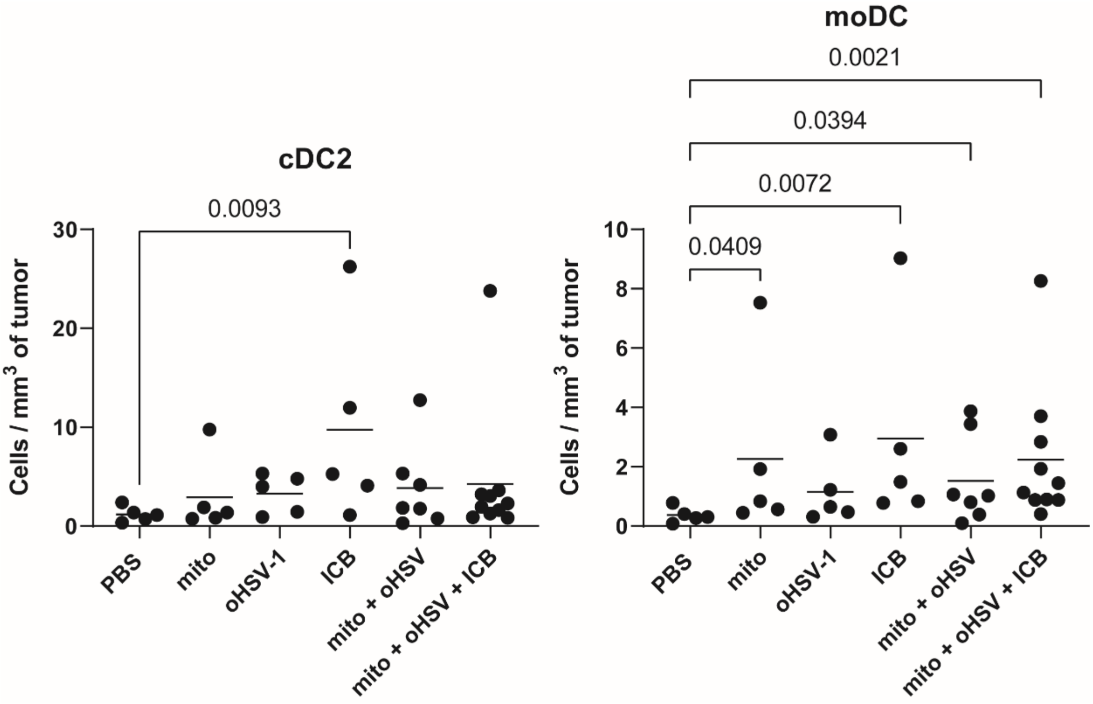

Figure S4. Tumor infiltration of cDC2s and moDCs. Mice bearing MC38 tumors were treated with different combinations of mito, oHSV and/or ICI. Tumors were harvested 4 days after start of treatment and the frequency of infiltrating immune cells was analyzed by flow cytometry. cDC2s (CD11c$^+$ MHCII$^+$ CD11b$^+$ Ly6C$^-$) and moDCs (CD11c$^+$ MHCII$^+$ CD11b$^+$ Ly6C$^+$) were graphed.

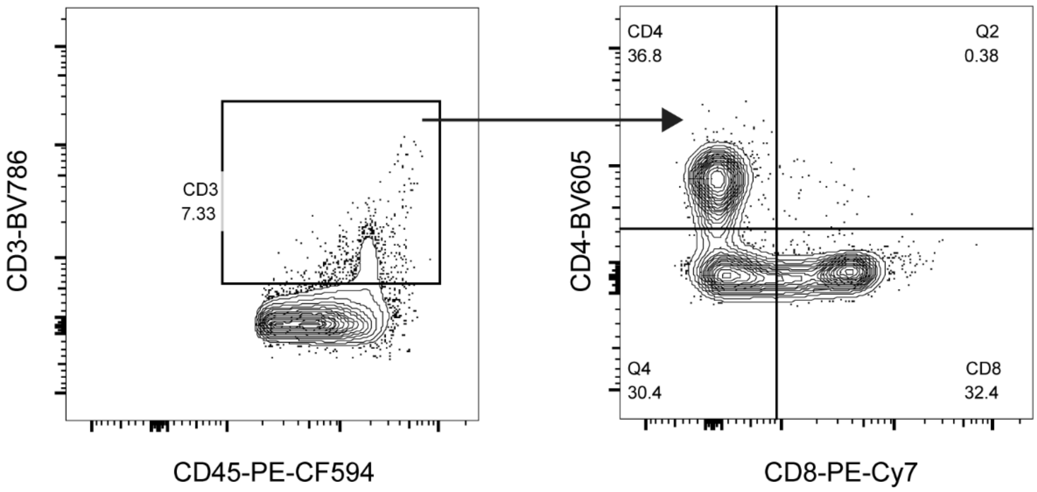

Figure S5. T **cell gating strategy.** Mice bearing MC38 tumors were treated with different combinations of mito, oHSV and/or ICI. Tumors were harvested 7 days after start of treatment and the frequency of infiltrating immune cells was analyzed by flow cytometry. Cells were gated on viable CD45$^+$.

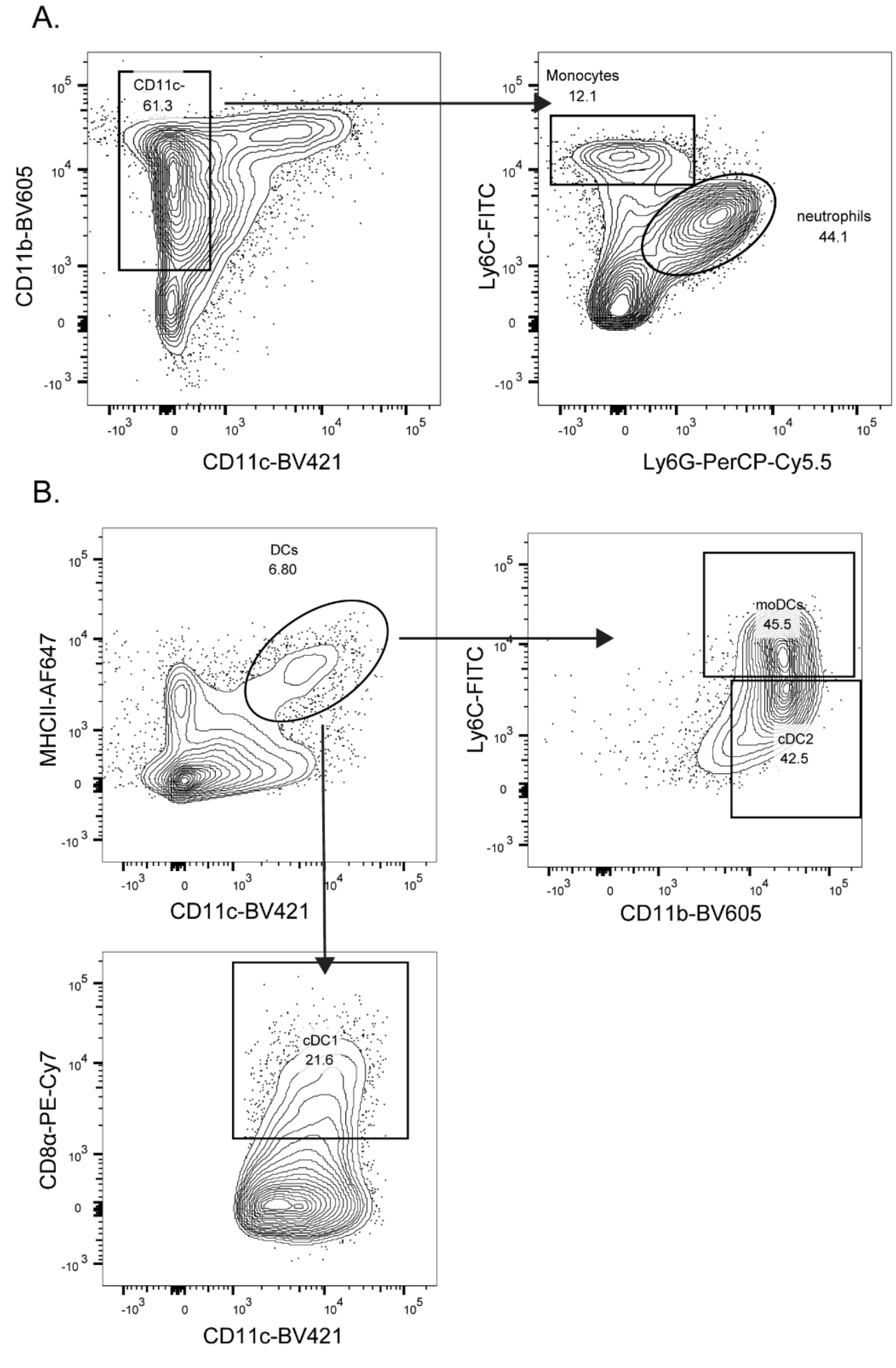

Figure S6. Myeloid gating strategy. Mice bearing MC38 tumors were treated with different combinations of mito, oHSV and/or ICI. Tumors were harvested 4 days after start of treatment and the frequency of infiltrating immune cells was analyzed by flow cytometry. Cells were gated on viable CD45^{+}. **(A)** Neutrophils and monocytes. **(B)** DCs.

Table S1. Differentially expressed genes associated with myeloid subset recruitment and activation. M = mito, O = oHSV, I = ICI. ns = P > 0.05.

Gene Symbol	M + O + I vs PBS	M + O vs PBS	ICI vs PBS	oHSV vs PBS	mito Vs PBS
Lcn2	14.18	11.69	2.52 (ns)	16.79	2.03 (ns)
Cxcl2	11.77	23.53	2.36 (ns)	8.38	1.96
Ccl3	9.78	16.41	3.17	8.46	1.43
Nos2	7.73	4.83	1.19	3.47	1.08
Serpinb2	7.55	3.04	2.35 (ns)	3.88	-1.13
S100a9	6.76	5.87	1.81	6.37	1.49
S100a8	6.62	7.54	2.36	5.99	1.24
Irg1	6.6	3.57	1.02	4.6	1.68
Ly6c2	5.81	5.08	4.02	5.85	2.41
Slfn4	5.8	7.46	1.39	3.33	2.37
Sell	5.24	5.23	2.03	4.85	2.04 (ns)
Ly6c1	4.26	2.66	3.24	3.43	1.65
Il1a	4.21	4.15	2.36	2.67	-1.15
Clec4d	3.85	3.54	3.24	3.05	1.79
Tarm1	3.69	1.91	5.02	1.89	1.69
Upp1	2.99	2.08	3.95	2.47	2.11
Il1b	2.96	2.24	1.75	2.74	-1.21
Cxcl1	2.92	1.84	1.65	1.72	-1.63
Ccl7	2.74	1.88	1.18	1.9	1.43
Ccrl2	2.63	2.53	2.79	2.09	1
Ccl5	2.47	3.11	1.73	1.34	2.77
Cxcl10	2.41	2.18	3.54	2.16	1.32
Cxcl5	2.01	1.38	1.88	1.69	-1.1
